# Associations between dietary patterns and anaemia in 6- to 23-month-old infants in central South China

**DOI:** 10.1186/s12889-021-10699-8

**Published:** 2021-04-09

**Authors:** Shao-hui Zou, Yuan Liu, Ai-bing Zheng, Zhi Huang

**Affiliations:** 1 Clinical Laboratory, The First People’s Hospital of Huaihua, Huaihua, Hunan China; 2 Mayang Maternal and Child Health Care Hospital, Huaihua, Hunan China; 3 Hunan University of Medicine, No. 492 Jinxi South Road, Huaihua, 418000 Hunan China

**Keywords:** Dietary pattern, Anaemia, Infant

## Abstract

**Background:**

Anaemia is prevalent in children. Therefore, this study examined the association between dietary patterns and anaemia among children in central South China.

**Methods:**

Cross-sectional studies were conducted in Mayang, central South China, in 2015 and 2018. Diet data were collected using a questionnaire, and dietary patterns were identified via exploratory factor analysis. Haemoglobin was measured to assess anaemia status. Associations between dietary patterns and anaemia were assessed using a logistic regression model.

**Results:**

The mean age of the infants surveyed was 14.06 months in 2015 and 16.58 months in 2018. Four dietary patterns were identified among infants aged 6–23 months: a diversified diet consisting mainly of tubers, dairy products, beans and bean products; a traditional diet consisting mainly of cereals, water, soup, vegetables and fruit; mainly breast milk, with a little powdered formula; or mainly multi-nutrient powders. The prevalence of anaemia in infants decreased from 29.49% in 2015 to 20.26% in 2018.In infants fed a diversified diet or multi-nutrient powders with top-quartile (Q4) scores, the risk of anaemia was reduced by 45%(adjusted odds ratio [AOR] = 0.55, 95%CI0.30–0.99, *P* = 0.047) or 59% (AOR = 0.41, 95% CI0.22–0.78, *P* = 0.006), respectively, compared to infants in the lowest quartile (Q1). Infants fed mainly breast milk had a 3.26-fold greater risk of anaemia compared to those with Q1 scores (AOR = 3.26, 95% CI 1.83–5.81, *P* < 0.001).

**Conclusions:**

Four dietary patterns were identified among infants aged 6–23 months in central South China. Infants should be fed a variety of food groups to improve their anaemia status.

## Background

Anaemia is prevalent in children and has short- and long-term effects on health and development, such as increasing the rate of growth retardation,decreasing immunity and intelligence, and even affecting an individual’s health in adulthood [1]. In 2011, 18.1% of children under 5 years of age worldwide were anaemic [2]. In China, the prevalence of anaemia in 2012 was 12.6% among children younger than5 years, and 20.5–28.2% in children aged 6–23 months [3].

Children grow and develop most vigorously in infancy, and insufficient iron intake from the diet is the main cause of anaemia in infancy [4–6]. The interactions of different foods with iron absorption are one reason for anaemia. For example, less iron is absorbed from plant foods because phytic and oxalic acids in plants can form insoluble iron compounds, thus reducing iron absorption [1]. However, most previous studies focused on the associations between individual foods or nutrients and anaemia in infants [4] and neglected the potential interactions between total dietary intake and foods or nutrients.

Dietary pattern analysis is becoming popular for evaluating associations between diet and health [7, 8]. A Singaporean study reported four dietary patterns among infants aged 6–12 months: predominantly breast milk, a diet based on guidelines, easy-to-prepare foods, and noodles and seafood [9]. Other studies have shown that dietary patterns among infants are associated with weight [10], the incidence of caries [11], bone mass [12], and the intelligence quotient [13]. However, few studies have clarified the dietary patterns related to anaemia in infants.

In this study, we identified dietary patterns among infants aged 6–23 months in central South China and clarified the associations between dietary patterns and anaemia.

## Methods

### Study design

In 2015 and 2018, cross-sectional studies were conducted in Mayang, an autonomous county associated with an ethnic minority group in central South China. The county has 18 towns or communities; there were 4698 births in 2015 and 3988 in 2018.The study enrolled caregivers with children aged 6–23 months in five Mayang communities. The minimum sample size was calculated using the following formula:

$$ n=\frac{{u^2}_{\alpha /2}\pi \left(1-\pi \right)}{\sigma^2} $$

According to the Report of Nutrition Development for Children Aged 0–6 Years in China [3], the prevalence of anaemia among children aged 6–23 months was approximately 25%. Therefore, in this study, π = 0.25, σ = 5%, α_1/2_ = 0.05, u = 1.96 and *n* = 288.The target sample size was increased from 288 to 300 after considering the likely dropout rate.

Using multistage random sampling, 312 and 311 children aged 6–23 months were recruited in 2015 and 2018, respectively. In 2015, five communities in Mayang were randomly selected according to the total number of births in the previous year. Then, five villages were randomly selected in each community according to the total number of births over the last year, or 25 villages in total. Lastly, proportional allocation was performed based on the total number of children aged 6–23 months, and 10–15 children aged 6–23 months were randomly selected in each village based on their age. The same sampling method was used in 2018.The numbers of births were obtained from the Mayang annual health report on mothers and children. Information on infants aged 6–23 months was obtained from the local mother-and-child healthcare system.

### Dietary data

Using a questionnaire, caregivers were asked about their children’s consumption of 15 food types—as defined in a guide on infant feeding and nutrition in China—over the previous 24 h, as in our previous study [14, 15]. These foods included breast milk, powdered formulas, milk powder and fresh milk, water and soup, sugar water and drinks, cereals, tubers, dark leafy vegetables and fruits, other vegetables and fruits, meat, eggs, dairy products, beans and bean products, nuts and multi-nutrient powders. The frequency of feeding breast milk, powdered formulas, milk powder, fresh milk, or multi-nutrient powders in the previous 24 h was recorded, whereas the consumption of other foods was recorded in a binary manner—yes (1) or no (0). The food list was similar in both surveys. The dietary questionnaires were completed in interviews by trained investigators.

### Dietary pattern analysis

Exploratory factor analysis was performed to identify dietary patterns. To understand the dietary patterns in 2015 and 2018, we combined both datasets before running the exploratory factor analysis. Firstly, the frequencies of the 15 foods were analysed using the Kaiser–Meyer–Olk in statistic. Then, dietary patterns were identified based on the eigenvalue (> 1), screen plot, factor interpretability, and proportion of variance explained. The foods with factor loadings> 0.3 were considered to contribute significantly to the identified factors. Lastly, standardised scores that represent the sum of intakes of food weighted by their factor loadings were calculated for each child. A higher factor score for a given pattern indicated that the participant was associated more strongly with this dietary pattern. The factor scores were categorised into four quartiles (Q1–4), with Q1representing a weak association with the dietary pattern and Q4 representing a strong association [16].

### Dietary pattern assessment

Eight World Health Organisation (WHO) infant and young child feeding indicators were used to assess the quality of the dietary patterns [17]. Definitions and parameters of child-feeding associated with each indicator were used to disaggregate the data by specified age groups (6–23 months). The numbers of children who met the following four evaluation criteria were recorded: the minimum dietary diversity, minimum meal frequency, minimum acceptable diet and consumption of iron-fortified food the previous day. Children who met the minimum dietary diversity criterion consumed foods from four food groups the previous day. Children who met the minimum meal frequency criterion received solid, semi-solid or soft foods at least the minimum number of times the previous day. Children who met the minimum acceptable diet criterion consumed foods with at least the minimum dietary diversity and the minimum meal frequency the previous day. Iron-rich or iron-fortified foods included naturally iron-rich foods, foods that were specially designed for infants and young children and fortified with iron, or foods that were fortified at home with a product that contains iron.

### Anaemia assessment

A capillary blood sample was collected by pricking the children’s fingers. Haemoglobin (Hb) was measured via a microchemical reaction method in g/dL using a HemoCue 130 (HemoCue, Sweden). The cut-off point for anaemia for children aged 6–23 months was an Hb level < 11.0 g/dL [18].

### Other variables

The information collected for the infants included sex (boy or girl), age (6–11, 12–17 or 18–23 months), birth weight (normal or low), gestational age (term or premature) and the presence of fever or diarrhoea in the previous 2 weeks (yes or no). Caregiver information included caregiver type (parents or grandparents and others), education level (illiterate, primary, junior, or senior and above), occupation (homemaker or others) and ethnicity (Miao or Han and others). This information was collected using the dietary questionnaire.

### Statistical analyses

The data are expressed as numbers and percentages for categorical variables. Significant differences were assessed using the χ^2^-test. The associations between various factors and anaemia were assessed using a logistic regression model. First, bivariate logistic regression analyses were performed to assess the relationships between age, sex, birth weight, gestational age, episode of diarrhoea or fever in the previous 2 weeks, infant dietary pattern, and caregiver type, education level, occupation, and ethnicity. Then, factors associated with *P* values ≤0.10 in the bivariate analysis were included in the multivariate logistic regression model. Odds ratios (ORs) and 95% confidence intervals (CIs) were calculated to determine the strength of the associations. A *P* value < 0.05 was considered indicative of statistical significance. All analyses were performed using Statistical Product and Service Solutions 13.

## Results

The surveys in 2015 and 2018 included 312 and 311 children aged 6–23 months, respectively. The mean infant age was 14.06 months in 2015 and 16.58 months in 2018. Table 1 summarizes the characteristics of the infants and caregivers. Nearly 60% of the infants were boys. Over 95% had a normal birth weight and were born at term. Approximately 85% of the caregivers were ethnic Miao. There was a higher proportion (37.82%) of infants aged 6–11 months in 2015, whereas a higher proportion of infants (44.69%) were aged 17–23 months in 2018. The prevalence of fever and diarrhoea in the previous 2 weeks was higher in 2015 than in 2018.Compared to 2015, the proportion of parents as caregivers and the caregiver education level increased in 2018, whereas the proportion of caregivers working as homemakers decreased. The prevalence of anaemia was higher in 2015 (29.49%) than in 2018 (20.26%).

**Table 1:** The characteristics of the infant aged 6 to 23 months and their caregivers in central-south China

Characteristics	Total		2015 (*n* = 312)		2018 (*n* = 311)		*P*
	n	%	n	%	n	%	
Infant							
Sex							
Boys	366	58.75	183	58.65	183	58.84	0.962
Girls	257	41.25	129	41.35	128	41.16	
Age							
6-11 months	187	30.02	118	37.82	69	22.19	< 0.001
12-17 months	195	31.30	92	29.49	103	33.12	
18-23 months	241	38.68	102	32.69	139	44.69	
Birth weight							
Normal	594	95.35	299	95.83	295	94.86	0.562
Low birth weight	29	4.65	13	4.17	16	5.14	
Gestational age							
Term	597	95.83	303	97.12	294	94.53	0.105
Premature	26	4.17	9	2.88	17	5.47	
Fever in the previous 2 weeks							
No	487	78.17	208	66.67	279	89.71	< 0.001
Yes	136	21.83	104	33.33	32	10.29	
Diarrhea in the previous 2 weeks							
No	552	88.60	268	85.90	284	91.32	0.034
Yes	71	11.40	44	14.10	27	8.68	
Caregivers							
Groups							
Parents	364	58.43	170	54.49	194	62.38	0.046
Grandparents and others	259	41.57	142	45.51	117	37.62	
Educational level							
Illiteracy	140	22.47	84	26.92	56	18.01	< 0.001
Primary	233	37.40	169	54.17	64	20.58	
Junior	199	31.94	48	15.38	151	48.55	
Senior and above	51	8.19	11	3.53	40	12.86	
Occupations							
Homemakers	535	85.87	302	96.79	233	74.92	< 0.001
Others	88	14.13	10	3.21	78	25.08	
Ethnicity							
Han and others	96	15.41	47	15.06	49	15.76	0.811
Miao	527	84.59	265	84.94	262	84.24	
Anaemia							
No	468	75.12	220	70.51	248	79.74	0.009
Yes	155	24.88	92	29.49	63	20.26	

Table 2 shows that four dietary patterns identified in infants aged 6–23 months accounted for 47.86% of the explained variance. Pattern 1 (diversified diet) included various foods and was mainly characterised by a high consumption frequency of tubers, dairy products, beans and bean products, nuts, dark leafy vegetables and fruits, meat and eggs. Pattern 2 (traditional diet) was positively correlated with the intake of cereals, water and soup, other vegetables and fruits, meat and multi-nutrient powders, but negatively correlated with the intake of milk powder and fresh milk. Pattern 3 (mainly breast milk) was associated with high intake of breast milk, but low intake of powdered formulas, milk powder and fresh milk. Pattern 4 (mainly multi-nutrient powders) had high positive loadings for multi-nutrient powders, sugar water and drinks and powdered formulas but negative loadings for dark leafy vegetables and fruits and cereals.

**Table 2:** Factor loadings for dietary patterns among infants aged 6 to 23 months in central-south China

Food	Diversified pattern	Traditional pattern	Breast milk pattern	Multi-nutrient powders pattern
Breast milk			0.712	
Powered formulas			−0.687	0.312
Milk powder and fresh milk		−0.431	−0.375	
Water and soup		0.551		
Sugar water and drink				0.356
Cereals		0.567		−0.330
Tubers	0.703			
Dark leaf vegetables and fruits	0.546			−0.344
Other vegetables and fruits		0.493		
Meat	0.440	0.470		
Egg	0.369			
Dairy products	0.696			
Bean and bean Products	0.658			
Nut	0.642			
Multi-nutrient powders		0.389		0.593

Table 3 lists the number of children who met the WHO infant and young child feeding criteria by the four quartiles of each dietary pattern. Children in the top quartile (Q4) of Pattern 1met the minimum dietary diversity (*P* < 0.001) and minimum acceptable diet (*P* < 0.001) criteria at higher rates. Children in the top quartile (Q4) of Pattern 2met the minimum dietary diversity (*P* < 0.001), minimum acceptable diet (*P* < 0.001) and consumption of iron-fortified food(*P* < 0.001) criteria at higher rates. Children in the top quartile (Q4) of Pattern 3met the minimum dietary diversity (*P* < 0.05), minimum meal frequency (*P* < 0.05) and minimum acceptable diet (*P* < 0.05) criteria at higher rates. However, the children in the top quartile (Q4) of Pattern 4met the minimum dietary diversity (*P* < 0.05), minimum meal frequency (*P* < 0.05) and minimum acceptable diet (*P* < 0.001) criteria at lower rates but consumed iron-fortified food at a higher rate (*P* < 0.001).

**Table 3:** The frequency of World Health Organization infant and young child feeding criteria in the four quartiles of dietary patterns

	Total	Minimum dietary diversity		Minimum meal frequency		Minimum acceptable diet		Consumption iron-fortified food	
	n	n	%	n	%	n	%	n	%
Diversified pattern									
Q1	154	2	1.30**	91	59.09	2	1.30**	107	69.48
Q2	159	81	50.94	101	63.52	56	35.22	113	71.07
Q3	158	127	80.38	97	61.39	78	49.37	115	72.78
Q4	152	149	98.03	96	63.16	94	61.84	92	60.53
Traditional pattern									
Q1	155	60	38.71**	78	50.32	32	20.65**	86	55.48**
Q2	157	80	50.96	110	70.06	56	35.67	107	68.15
Q3	159	96	60.38	107	67.30	66	41.51	114	71.70
Q4	152	123	80.92	90	59.21	76	50.00	120	78.95
Breast milk pattern									
Q1	156	68	43.59*	87	55.77*	38	24.36*	106	67.95
Q2	156	103	66.03	87	55.77	54	34.62	114	73.08
Q3	155	101	65.16	104	67.10	70	45.16	104	67.10
Q4	156	87	55.77	107	68.59	68	43.59	103	66.03
Multi-nutrient powders pattern									
Q1	156	93	59.62*	108	69.23*	68	43.59**	57	36.54**
Q2	155	103	66.45	104	67.10	71	45.81	110	70.97
Q3	164	93	56.71	95	57.93	53	32.32	131	79.88
Q4	148	70	47.30	78	52.70	38	25.68	129	87.16

*P < 0.05, **P < 0.001; *P* for trend

Table 4 describes the associations between potential factors and anaemia among infants as shown by the logistic regression model. The risk of anaemia in 2018 decreased 14% compared to 2015 (adjusted OR [AOR] = 0.86, 95%CI0.74–1.00, *P* = 0.047). Children in the top quartile (Q4) of Pattern 1had a lower risk of anaemia (− 45%; AOR = 0.55, 95% CI 0.30–0.99, *P* = 0.047) compared to those in the lowest quartile (Q1). Children in the top quartile (Q4) of Pattern 4 saw their risk of anaemia reduced by 59% (AOR = 0.41, 95% CI 0.22–0.78, *P* = 0.006) compared to those in the lowest quartile (Q1). Children in the top quartile (Q4) of Pattern 3 had a 3.26-fold greater risk of anaemia compared to those in the lowest quartile (Q1) (AOR = 3.26, 95% CI1.83 to5.81, *P* < 0.001).

**Table 4:** The associations between potential factors and anaemia by the logistic regression model among infants age 6 to 23 months in central-south China

Factors	N	n	%	COR(95%CI)	*P*	AOR(95%CI)	*P*
Year							
2015	312	92	29.49	1		1	
2018	311	63	20.26	0.85 (0.75,0.96)	0.008	0.86 (0.74,1.00)	0.047
Diarrhea							
No	551	131	23.77	1		1	
Yes	71	24	33.80	1.64 (0.96,2.78)	0.068	1.76 (1.00,3.09)	0.050
Diversified pattern							
Q1	154	49	31.82	1		1	
Q2	159	42	26.42	0.77 (0.47,1.25)	0.293	0.83 (0.49,1.41)	0.487
Q3	158	39	24.68	0.70 (0.43,1.15)	0.162	0.98 (0.57,1.70)	0.957
Q4	152	25	16.45	0.42 (0.24,0.73)	0.002	0.55 (0.30,0.99)	0.047
Breast milk pattern							
Q1	156	26	16.67	1		1	
Q2	156	25	16.03	0.95 (0.52,1.74)	0.878	1.04 (0.56,1.94)	0.900
Q3	155	42	27.10	1.86 (1.07,3.22)	0.027	2.00 (1.12,3.58)	0.020
Q4	156	62	39.74	3.30 (1.94,5.60)	< 0.001	3.26 (1.83,5.81)	< 0.001
Multi-nutrient powders pattern							
Q1	156	44	28.21	1		1	
Q2	155	44	28.39	1.01 (0.62,1.65)	0.972	0.80 (0.47,1.37)	0.425
Q3	164	42	25.61	0.88 (0.53,1.44)	0.601	0.71 (0.40,1.26)	0.245
Q4	148	25	16.89	0.52 (0.30,0.90)	0.020	0.41 (0.22,0.78)	0.006

*: Only factors with a value of *P* ≤ 0.10 in a bivariate analysis were shown in the Table. N: The total; n: number of anaemia

1: Reference category. COR: crude odds ratio; AOR: adjusted odds ratio

**Fig. 1:**
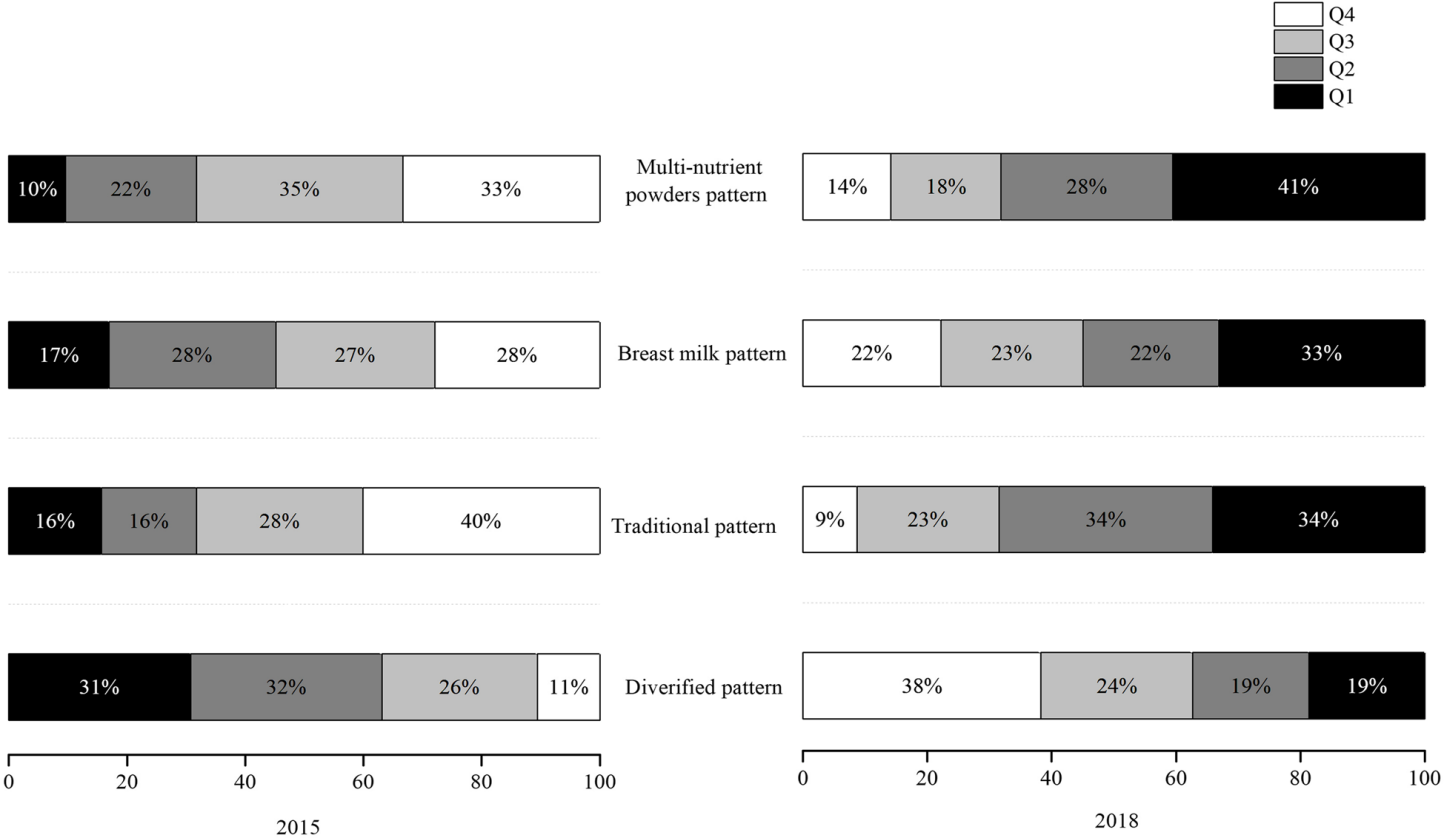
The constituent ratios among the four quartiles for each dietary pattern in 2015 and 2018

Figure 1 shows the constituent ratios among the four quartiles for each dietary pattern in 2015 and 2018. Significant differences in the constituent ratios among the four quartiles for the four dietary patterns were found in 2015 versus 2018.Moreover, higher proportions of children in the top quartiles (Q4) in terms of consuming a traditional (χ^2^ = 106.656, *P* < 0.001), multi-nutrient powder(χ^2^ = 103.045, *P* < 0.001),or breast milk (χ^2^ = 21.755, *P* < 0.001) diet were found in 2015, whereas a higher proportion of children in the top quartile (Q4) in terms of consuming a diversified diet was found in 2018(χ^2^ = 69.890, *P* < 0.001).

## Discussion

Our study identified four dietary patterns among infants aged 6–23 months in central South China. The diversified diet was characterised by high consumption of various foods. The traditional diet was positively correlated with the intake of cereals, water and soup, other vegetables and fruits, and meat. The breast milk diet was associated with a high intake of breast milk. The multi-nutrient-powder diet was associated with high positive loadings for multi-nutrient powders. The prevalence of anaemia among the infants decreased from 29.49% in 2015 to 20.26% in 2018. The diverse and multi-nutrient-powder diets were associated with a reduction in the risk of anaemia, whereas the breast milk diet was associated with increased risk.

The diversified diet included a variety of food groups and was characterised by high consumption of tubers, dairy products, beans and bean products, nuts, dark leafy vegetables and fruits, meat and eggs. Infants on this diet met the minimum dietary diversity and minimum acceptable diet criteria of the WHO infant and young child feeding guidelines at higher rates, thus this diet followed healthy guidelines or patterns, as reported in previous studies [11, 19]. The traditional diet was characterised by high intake of cereals, water and soup, other vegetables and fruits, meat and multi-nutrient powders, similar to the traditional Chinese adult diet [20–22]. The breast milk diet resembled the ‘breastfeeding’ pattern reported by Lim [9] and Smithers [23] and was characterised by a higher intake of breast milk and lower intake of formula milk. Lastly, the multi-nutrient-powder diet, which was characterised by high intake of multi-nutrient powders, sugar water and drinks, and powdered formulas, has not been recorded in other studies. This diet constitutes a special dietary pattern in central South China that may be associated with a free government micronutrient supplementation program called Ying Yang Bao, which has been operating since 2009 to improve children’s health in poor rural areas of China [24]. However, children on the multi-nutrient-powder diet met the minimum dietary diversity, minimum meal frequency and minimum acceptable diet criteria at lower rates. This implies that the feeding practices of infants need to be improved in the implementation of the Ying Yang Bao program.

The logistic regression model revealed that the diversified diet reduced the risk of anaemia, whereas the breast milk diet increased the risk. The diversified diet offers infants a variety of food groups. Different studies have shown that dietary diversity reduces the risk of malnutrition among infants and children [25, 26], and dietary diversity is promoted as a nutrition intervention in many areas [27]. The breast milk diet with a higher intake of breast milk increased the risk of anaemia among infants. Although breast milk is an ideal infant food, it contains relatively little iron and cannot meet the demands of rapid growth and development in children after the age of 6 months [28]. We found that the risk of anaemia decreased in children on the multi-nutrient-powder diet, indicating that the Ying Yang Bao program is effective at reducing anaemia in children in rural China [24, 29].

We also found that children were more likely to be anaemic in 2015 than in 2018. This may have been partly due to a change in food habits from 2015 to 2018. We found that a higher proportion of children adhered to the breast milk diet in 2015 versus a higher proportion adhering to the diversified diet in 2018.Consumption of the diversified diet reduced the risk of anaemia, whereas the predominant consumption of breast milk increased the risk. The decreasing prevalence of anaemia from 2015 to 2018 may also have been attributed to the higher proportion of children aged 18 to 23 months in 2018. Previously, we demonstrated that the risk of anaemia was higher among children aged 6–12 months compared to children aged 18–23 months [14].

One strength of this study is that we collected data in two different years in cross-sectional surveys. The study identified four dietary patterns and their associations with anaemia in infants. Limitations of our study include the fact that cross-sectional designs cannot fully eliminate recall error with regard to questionnaire information. Furthermore, foods high in oils and salt as well as snacks were excluded; thus food intake could not be assessed accurately in our dietary survey. Lastly, all of the participants were from rural areas and do not represent the general population of children in China, which may limit the generalisability of our findings.

## Conclusions

Four dietary patterns were identified among infants aged 6–23 months in central South China. Consumption of the diverse and multi-nutrient-powder diets reduced the risk of anaemia. However, children on the multi-nutrient-powder diet met the minimum dietary diversity, minimum meal frequency and minimum acceptable diet criteria at lower rates. Micronutrient supplementation may be an effective measure to reduce the risk of anaemia among infants aged 6–23 months in central South China, but infant feeding practices should be improved.

## Data Availability

The datasets used and/or analyzed during the current study are available from the corresponding author on reasonable request.

## Abbreviations

Hb: Hemoglobin
n: Number
OR: Odds Ratio
CI: Confidence Interval
YYB: Ying Yang Bao
